# Self-perceived quality of sleep among COPD patients in Greece: the SLEPICO study

**DOI:** 10.1038/s41598-021-04610-z

**Published:** 2022-01-11

**Authors:** Nikolaos Koulouris, Katerina Dimakou, Konstantinos Gourgoulianis, Nikolaos Tzanakis, Aggeliki Rapti, Mina Gaga, Niki Georgatou, Paschalis Steiropoulos, Christos Karachristos, Athena Gogali, Konstantinos Kalafatakis, Konstantinos Kostikas

**Affiliations:** 1 First Department of Pulmonary Medicine and Intensive Care Unit, National and Kapodistrian University of Athens, Medical School, 115 27 Athens, Greece; 2 5Th Respiratory Medicine Department, General Hospital for Chest Diseases of Athens “SOTIRIA”, Athens, Greece; 3 Department of Respiratory Medicine, Faculty of Medicine, University of Thessaly, BIOPOLIS, 41500 Larissa, Greece; 4 Department of Respiratory Medicine, University General Hospital of Heraklion, Medical School, University of Crete, 71003 Heraklion, Greece; 5 2Nd Respiratory Medicine Department, General Hospital for Chest Diseases of Athens “SOTIRIA”, Athens, Greece; 6 7Th Respiratory Medicine Department, Athens “Sotiria” Chest Diseases Hospital, Athens, Greece; 7 Iatriko Athinon, Areos 41, Paleo Faliro, Greece; 8 Department of Respiratory Medicine, Medical School, University General Hospital of Alexandroupolis, Democritus University of Thrace, Alexandroupolis, Greece; 9 Department of Pulmonary Medicine, General Hospital of Thessaloniki “Georgios Papanikolaou”, G. Papanikolaou Ave, 57010 Exohi, Greece; 10 Department of Informatics and Telecommunications, School of Informatics and Telecommunications, University of Ioannina, Arta, Greece; 11 Respiratory Medicine Department, University Hospital of Ioannina, Ioannina, Greece

**Keywords:** Health care, Medical research, Signs and symptoms

## Abstract

Chronic obstructive pulmonary disease (COPD) is a leading cause of morbidity and mortality worldwide accompanied by a substantial social and economic burden for the patient and the society. Poor sleep quality among COPD patients is frequently unnoticed and unaddressed by physicians and patients themselves, although it is a major source of further deterioration of these patients’ quality of life. The aim of the present study was to record the quality of sleep in COPD patients among the Greek population and correlate these findings with various features of these patients, using the COPD and Asthma Sleep Impact Scale (CASIS). This was a cross-sectional observational study. Forty different variables (demographics, vital sign measurements, COPD-related medical history parameters, comorbidities, CASIS questionnaire results, COPD assessment test, COPD severity based on spirometry measurements, COPD stage based on the ABCD assessment approach, inhaled COPD treatment report) were collected from 3454 nation-wide COPD patients (Greece). The study sample consisted of COPD patients, mainly male (73%) with a median age of 69 years and a median BMI of 27.2. More than half of COPD patients (60.6%) suffered from moderate disease severity and 23.8% from severe disease, while less than half (42.1%) suffered from at least one exacerbation of the disease over the last year prior study enrollment. About 14% reported frequent to very frequent issues affecting their sleep quality, between a fourth and a third of them reported occasional night sleep disturbances, and at least half of them reported no or very infrequent problems in their night sleep. Our study indicates that the COPD assessment test (CAT) and the spirometry-based disease severity can predict the poorness in the quality of sleep (F_2,3451_ = 1397.5, p < 0.001, adj. R2 = 0.45) as assessed by CASIS score, and that the latter also correlates with age (ρ = 0.122, p < 0.001) and disease duration (ρ = 0.104, p < 0.001). On the contrary, there appears to be no correlation between sleep quality and number of exacerbations. Finally, untreated patients with COPD suffer from poorer quality of sleep compared to treated subjects, independently of the use of inhaled corticosteroids (F_2,3451_ = 21.65, p < 0.001). The results of the SLEPICO study show that increased age, prolonged disease duration, and especially CAT score ≥ 10, and severe COPD stage, might act as important indicators for deterioration in the quality of sleep, with potential consequences in the daily routine of those patients, thus urging potentially for further pharmacological interventions or modifications.

## Introduction

Chronic obstructive pulmonary disease (COPD) is a leading cause of morbidity and mortality worldwide accompanied by a substantial social and economic burden for both the patients and the society^1^. Aside the main symptoms, deriving from the underlying respiratory dysfunction (i.e., cough, dyspnea, increased sputum production), sleep impairment has also been widely recognised as a common problem in COPD patients attributed to a multifactorial process, involving physiological changes associated with sleep, disturbance in gas exchanges, and/or COPD medications^2^. Changes in breathing patterns during sleep, that often do not compromise healthy individuals’ ventilation and gas exchange, may result in significant hypoxemia and hypercapnia in COPD patients. The underlying pathophysiology of sleep-related disturbances in the context of COPD includes a decrease in respiratory drive, respiratory muscle hypotonia, increased airway resistance and an increased functional residual capacity. These changes lead to hypoventilation and ventilation/perfusion mismatch resulting in worsening hypoxemia and/or emergence of hypercapnia^2,3^. As a result of these mechanisms, COPD patients often have difficulty in initiating or maintaining sleep, experience shorter duration of REM sleep periods and increases in light sleep and frequent arousals and sleep fragmentation^4,5^.

Poor sleep quality and night-time symptoms are often under-reported by COPD patients or unnoticed and unaddressed by physicians, even though the detrimental effects of sleep deprivation in the overall well-being and functioning of individuals are well documented^6–9^. Sleep restriction has been found to negatively affect neurocognitive functions^10^, the cardio-metabolic homeostasis^11^ and is linked to weight gain, altered endocrine responses and accidents^12^.

Several questionnaires have been utilized to measure and assess sleep quality and sleep disorders. Recent systematic reviews have identified various patient-reported outcome measures (PROs) that have been applied in the context of COPD^13,14^. Among them, the two most frequently used are the Epworth Sleepiness Scale and the Pittsburgh Sleep Quality Index, although they are not disease-specific nor have been validated for use in COPD patients. A PRO fulfilling these requirements is the COPD and Asthma Sleep Impact Scale (CASIS)^15^. The aim of the present study was to assess the quality of sleep in COPD patients among the Greek population, using the CASIS questionnaire, and correlate these findings with various features of these patients and, finally, provide initial evidence on the role of COPD treatment in modifying quality of sleep, as reported by patients.

## Materials and methods

### Bioethical considerations

The study was conducted according to the provisions of Good Clinical Practice (GCP), local laws, EU-Directive 2001/20, and the International Conference on Harmonization and the World Medical Association Declaration of Helsinki guidelines. Prior to study initiation, study protocol and all relevant documentation was submitted to and approved by the Institutional Review Board/ Ethics Committees (IRB/EC) of the following institutions: “Sismanogleio-Amalia Fleming” General Hospital of Attiki, “Sotiria” Athens General Hospital for Thorax Diseases, “Metropolitan” Hospital of Athens, University General Hospital of Heraklion, “Evangelismos” General Hospital of Athens, “G. Papanikolaou” General Hospital of Thessaloniki, “Euromedica” Hospital of Thessaloniki, University General Hospital of Ioannina, University General Hospital of Larisa, University General Hospital of Patra, “Iatriko” General Hospital (P. Faliro, Attiki), University General Hospital of Alexandroupolis, General Hospital of Kavala. Given the sensitive nature of data processed in the frame of the study, all parties involved undertook adequate safety measures (physical, logical, organisational, technical, etc.) to warrant that data would always be processed safely and in compliance with the EU Data Privacy Directive 95/46/EC.

### Participants

Patients fulfilled the following inclusion criteria for inclusion in the study: (i) male and female aged ≥ 40 years, (ii) established (i.e., under treatment for at least a month) or new diagnosis of COPD according to 2017 GOLD recommendations, (iii) absence of exacerbations neither at baseline visit nor in the previous month (i.e., have a stable COPD), (iv) willingness and ability to give informed consent prior to study enrollment, and compliance with study procedures. Exclusion criteria were: (i) pregnancy or lactation (in case of female subjects), (ii) a previous diagnosis of asthma, sleep disordered breathing (SDB) or other chronic respiratory disease other than COPD, (iii) any acute or chronic condition that would limit the patient’s ability to complete questionnaires or participate in the study (iv) participation in another study. Subjects were able to withdraw from the study at any time without explanation and without losing the right to receive medical care.

### Study objectives

The primary objective of the present study was to record the quality of sleep in COPD patients among the Greek population. Secondary objectives include (i) correlating the quality of sleep in COPD patients with the severity of the disease, according to the Global Initiative for Chronic Obstructive Lung Disease (GOLD) 2017 recommendations and (ii) providing evidence on the role of COPD-related inhaled therapy in modifying quality of sleep, as reported by patients.

### Study structure and workflow

This was a multicenter, observational, non-interventional study that involved a single study visit for each participant. A total of 3454 participants had been included. The study took place in 308 different healthcare centers across Greece and lasted for 17 months, between February 2018 and July 2019. To reduce any potential selection bias, recruitment sites were requested to ensure consecutive assessment of all potential eligible patients and consequently enroll in this manner. The recruitment process included the signing of a consent form by the patient which was followed by a complete medical examination. Then, the investigators documented any potential adverse events until the study was over. In addition, all patient data had been recorded in an electronic Case Report Form (eCRF). This form included the outcome measures described below. Each study investigator had the ultimate responsibility for the collection and for reporting all clinical, safety and laboratory data entered on the eCRF and any other data collection forms (source documents) and ensuring that they were accurate, authentic/original, attributable, complete, consistent, legible, and available when required. Data entry and locking was done automatically at the site level, by properly authorized personnel (either the principal investigator or delegated site staff), to assure the quality of the collected data (Supplementary Fig. 1).

### Study assessments

#### Demographics

The body weight, height, and gender of the participants was recorded.

#### Vital sign measurements

Diastolic and systolic blood pressure along with heart rate has been measured and recorded by the responsible physicians.

#### COPD medical history

Patients were asked to provide information regarding their COPD medical history. This included the time of first diagnosis (i) any current medication (ii) and their family history (iii). Furthermore, a GOLD 2017 classification of the disease was established by the responsible physician (iv).

#### Spirometric measurements

Forced expiratory volume in 1 s (FEV_1_) and forced vital capacity (FVC) measurements had been undertaken and noted in the eCRF forms for each participant.

#### CASIS Questionnaire

A Greek version of the CASIS 7-item questionnaire has been used in the study. The questionnaire consists of two parts. The first part includes five questions, determining the frequency that patients had a poor night sleep during the previous week. Each question examines this topic from slightly different perspectives; (overall) bad night’s sleep, sleepiness during the day, difficulty falling asleep, waking up during the night with breathing problems, difficulty reestablishing sleep after waking up during the night. The last two questions approach the topic from the exact reverse perspective (frequency of overall good night sleep and frequency of waking up in the morning feeling rested during the previous week). Questions are scored on a Likert scale ranging from 1 (= never) to 5 (= very often), referring to occurrence of symptoms. For each subject, the scores of questions 6–7 were reversed, then scores of questions 1–7 linearly transformed to a 0–100 scale, and finally averaged between each other, to give a final score, reflecting the poorness of each subject’s night sleep. High scores indicate a major impact of the COPD on the patient’s sleep^15^.

#### Comorbidities

Patients provided information about any underlying comorbidities and how they were being treated.

#### COPD assessment test (CAT)

The patients were asked to fill a CAT questionnaire onsite. Again, results were reported into the eCRF form. The CAT consists of eight items (cough, phlegm, chest tightness, breathlessness, limited activities, confidence leaving home, sleeplessness and energy). Item scores range from 0 to 5 points resulting in a CAT total score ranging from 0 to 40 points. The minimal clinically important difference of the CAT is 2 points^16^.

#### 38Classification of subjects based on the disease spirometric severity and GOLD 2017 ABCD classification

Lung function (spirometric) data, recorded by the investigator by means of FEV1 (Forced Expiratory Volume in 1 s) and FVC (Forced Vital Capacity), pre- and post-bronchodilation, according to common clinical practice, and the severity of airflow limitation was determined as mild (post-bronchodilation FEV_1_ ≥ 80% predicted), moderate (post-bronchodilation FEV_1_ = 50–79% predicted), severe (post-bronchodilation FEV_1_ = 30–49% predicted) and very severe (post-bronchodilation FEV_1_ < 30% predicted). Moreover, patients were asked about their disease exacerbation history (frequency, severity) over the previous 12 months, and were subsequently classified as belonging to Group A (CAT score < 10, up to one exacerbation not leading to hospitalization), B (CAT score ≥ 10, up to one exacerbation not leading to hospitalization), C (CAT score < 10, 2 + exacerbations or any leading to hospitalization) or D (CAT score ≥ 10, 2 + exacerbations or any leading to hospitalization) of the ABCD assessment tool suggested by GOLD 2017 guidelines^17^.

#### Inhaled COPD treatment report

During the study, the patients were asked to complete a subjective treatment report, if applicable, stating as to whether the treatment was successful according to their subjective opinion and experience (Yes or No question).

### Statistical analysis

All variables were summarized by frequency distribution tables (categorical variable) or are expressed as in the number of cases. Their median, mean, standard deviation (SD), or range for continuous variables, has been calculated. Missing values have not been replaced and all analysis is based on the number of existing values from any part of the sample. Any missing values occurred in a random manner i.e., could not introduce a systematic bias into the study. Prior to any statistical comparisons or investigating any associations, data have been checked for being normally distributed, independence and normal distribution of residuals, linear relationship between CASIS scores, CAT scores and spirometric severity scores, equal error variances, absence of any multicollinearity or significant number of outliers. Associations between CASIS questionnaire, spirometric severity and CAT questionnaire values (with and without its sleeplessness component) have been investigated by multiple linear regression analysis. Moreover, associations between CASIS and COPD disease stage, ABCD groups, age of patients and time-since-diagnosis have been investigated by Spearman rank correlation coefficient ρ. Finally, associations between CASIS and the type of therapeutic approach (no treatment, treatment involving inhaled corticosteroids, other treatments) per CAT score subcategory has been investigated by analysis of variance (ANOVA). All tests were 2-sided, and the significance level p was set to 0.05. Bonferroni correction has been applied to account for multiple comparisons.

## Results

### Population sample characteristics

3454 COPD patients participated in the study. Male to female ratio was just below 3:1 in our study sample (male participants 73%) with an age ranging from 40 to 97 years (mean age 68.5, median age 69 years, SD 9.7 years). More than 80% of our study sample belonged to the 60 + years of age group. The mean (and median) body mass index (BMI) was higher than 27 (with a SD of 4.8) with more than 70% of our study sample belonging to the overweight or obese BMI group. Only fifty-five subjects (1.6%) were underweight to severely underweight. Furthermore, the mean systolic/ diastolic blood pressure (± SD) was 130/79 (12.2/8.7), the mean heart rate (± SD) 78 (9.1) and the mean oxygen saturation (± SD) 95% (2.8). The majority of our population sample didn’t suffer from any disease exacerbations over the last 12 months preceding study enrollment (57.9%), about a fifth suffered from 1 exacerbation (22.6%) and about another fifth from 2 or more exacerbations (19.5%) (Table 1). Almost three quarters (74%) of the COPD patients were diagnosed more than a year ago, a fifth of them (19%) within the last 6 months, and just the remaining 7% between 6–12 months. Only a 13.4% of the patients had a family history of COPD. The great majority of subjects belonged to the moderate (61%) or severe stage (24%) of the disease, and there were 4.5 times more patients suffering from more symptomatic disease (CAT score ≥ 10, i.e., groups B, D) than patients with milder symptomatology (CAT score < 10, i.e., groups A, C). Treatment with one pharmaceutical product, LAMA/LABA combination, was the most popular COPD management strategy (45% in this sample), while a LAMA and/or LABA agent (either as monotherapy or included in combination treatments) was prescribed in 64% of the study participants. The most frequent comorbid conditions, present in our population sample, were cardiovascular disorders (36.4%) followed by metabolic disorders (10.4%) (Supplementary Fig. 2).

**Table 1:** Summary statistics of the demographic features, vital signs, and COPD characteristics of the study sample. CAT: COPD assessment test, COPD: chronic obstructive pulmonary disease, ER: emergency room/ department, FEV1: the vital capacity that a person can expire in the first second of forced expiration, SaO_2_: blood oxygen saturation, YoA: years of age.

**Gender (N = 3454)**			
Male, n (%)			2521 (73%)
**Age (years) (N = 3454)**			
Median (25–75%)	69 (62–75)	Range	40–97
**Age group**			
40–60 YoA	625 (18.1%)		
≥ 60 YoA	2829 (81.9%)		
**BMI (kg/m**^**2**^**) (N = 3454)**			
Median (25–75%)	27.2 (24.6–30.7)	Range	15.2–52.4
**BMI group**			
< 18.5 kg/m^2^	55 (1.6%)		
18.5 – 24.9 kg/m^2^	924 (26.7%)		
25 – 29.9 kg/m^2^	1484 (43%)		
> 30 kg/m^2^	991 (28.7%)		
**Systolic/Diastolic blood pressure (mmHg) (N = 3447)**			
Median (25–75%)	130/80 (120/70–140/85)	Range	210/120–85/45
**Heart rate (bpm) (N = 3454)**			
Median (25–75%)	78 (70–84)	Range	42–140
**SaO**_**2**_** at rest (%) (N = 3452)**			
Median (25–75%)	95 (94–96)	Range	62–99
**COPD severity (FEV**_**1**_** post-bronchodilation % of predicted) (N = 3450)**			
Very Severe (< 30%)			148 (4.3%)
Severe (30–49%)			821 (23.8%)
Moderate (50–79%)			2092 (60.6%)
Mild (≥ 80%)			389 (11.3%)
**COPD exacerbations (N = 3454)**			
Number of patients with 1 + exacerbation(s) in the previous 12 months			1455 (42.1%)
**Quantity and quality of exacerbations (N = 1455)**			
Median number of exacerbations (25–75%)	1 (1–2)	Range	1–8
Number of patients with exacerbations that led them to the ER (and number of such type of exacerbations per patient)			260(1) 53(2) 12(3) 2(4)
Number of patients with exacerbations that led them to hospitalization (and number of such type of exacerbations per patient)			174(1) 26(2) 5(3) 1(4)
**CAT total score (N = 3454)**			
Median (25–75%)	16 (11–21)	Range	0–40

### CASIS questionnaire

**Figure 1:**
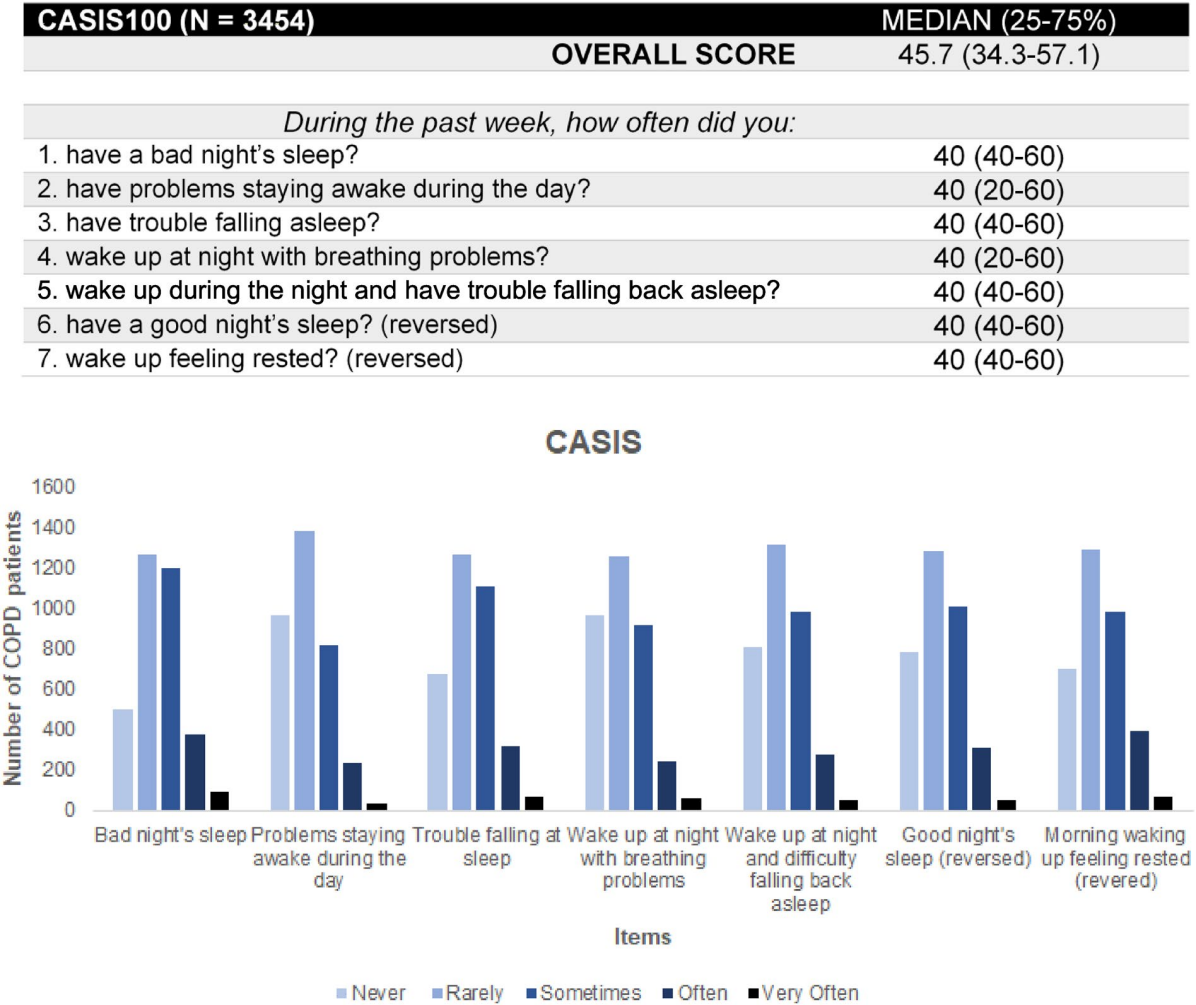
(Upper panel) Summary statistics of the overall score and the score from each of the 7 items of the CASIS questionnaire. (Lower panel) Distribution of the responses of the study participants in each of the 7 items of the CASIS questionnaire. To present all data in a uniform manner (in that the directionality of best-to-worst responses is always left-to-right), the values in the last two items have been reversed. CASIS: COPD and Asthma Sleep Impact Scale, CASIS100: COPD and Asthma Sleep Impact Scale, linearly transformed to a 0–100 scale, COPD: chronic obstructive pulmonary disease.

patients responded, on average, in a similar pattern across all 7 items of the sleep quality questionnaire. In particular, 8–14% reported often to very often sleep disturbances, 24–35% of them reported occasional night sleep disturbances and 51–68% of all patients reported no or very infrequent problems in their night sleep (Fig. 1). The great majority of patients under treatment (87%) subjectively reported that sleep quality has improved following the initiation of the current inhaled COPD treatment (Supplementary Fig. 3).

### Correlations between the CASIS questionnaire and population sample characteristics

**Figure 2:**
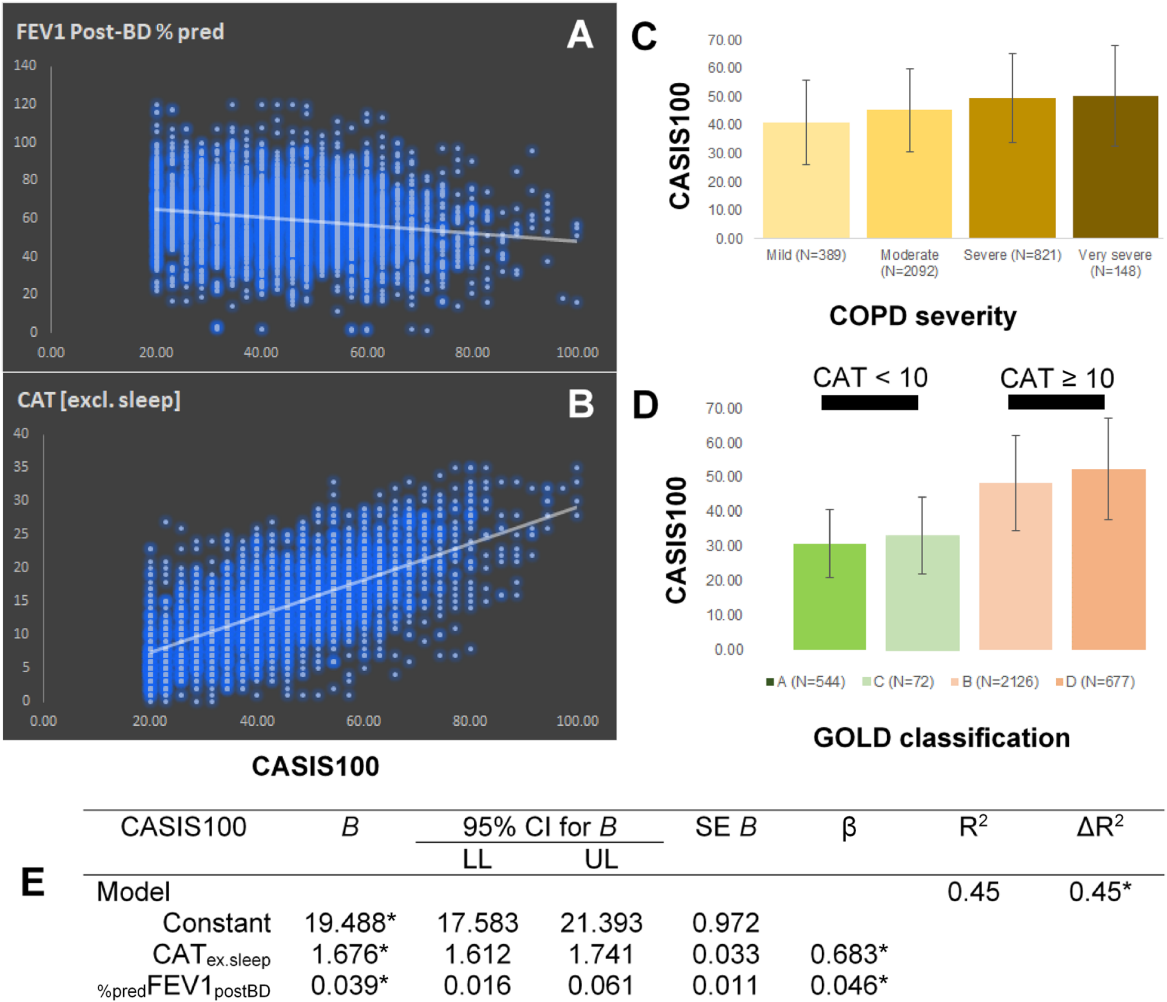
(**Α**) Plotting the %predicted FEV1 spirometric data post-bronchodilation (FEV1 Post-BD % pred or %predFEV1postBD) against the CASIS100 scores show a negative linear correlation (ρ = −0.180, *p* < 0.001). (**B**)Plotting the total CAT score excluding its sleeplessness component (CAT[excl. sleep] or CATex.sleep) against the CASIS100 scores show a positive linear correlation (ρ = 0.668, p < 0.001). (**C**) As the disease severity increases, patients tend to have higher CASIS scores, i.e., experience poorer quality of sleep. (**D**) Patients belonging to groups B and D of the GOLD ABCD classification system have notably poorer quality of sleep compared to those belonging to groups A and C. (**E**) Multiple regression results for CASIS100, using as independent variables %predFEV1postBD and CATex.sleep. B: unstandardized regression coefficient, CASIS100: COPD and Asthma Sleep Impact Scale, linearly transformed to a 0–100 scale, CAT: COPD assessment test, CI: confidence intervals, COPD: chronic obstructive pulmonary disease, GOLD: Global Initiative for Chronic Obstructive Lung Disease, LL: lower limit, UL: upper limit, SE B: standard error of the coefficient, β: standardized coefficient, R2: coefficient of determination, ΔR2: adjusted R2 *p ≤ 0.001.

**Figure 3:**
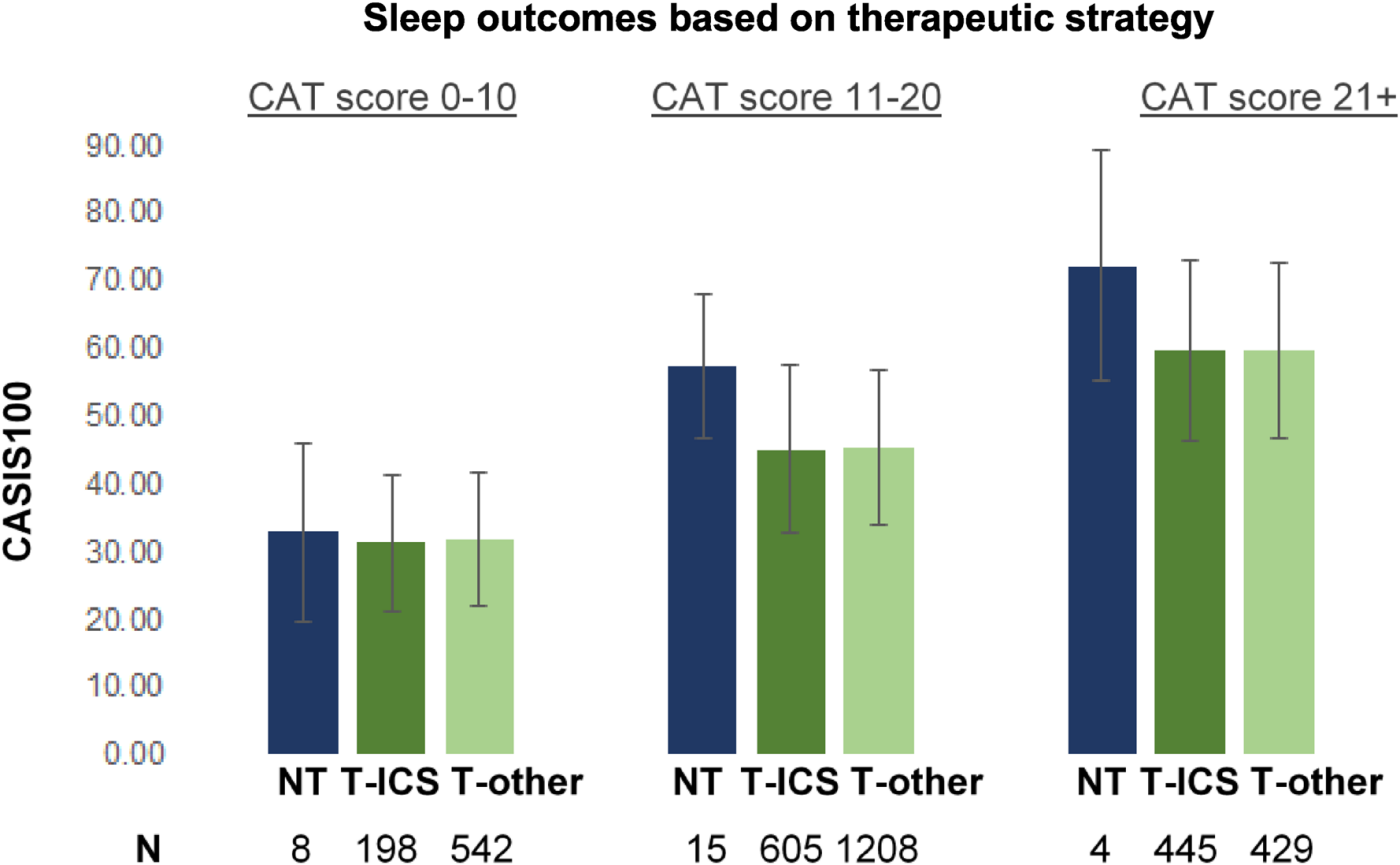
Sleep outcome, measured by CASIS, based on the different therapeutic approaches, and adjusted to different ranges of CAT score. As we have established so far (see main text and previous figures), sleep outcomes in COPD patients correlate moderately with their age, TsD and disease severity stage, and strongly with the symptoms of the disease (estimated by CAT). For low CAT scores (0–10), treatment or no treatment does not seem to impact sleep outcomes. Nevertheless, for higher CAT scores (11 +), any treatment seems to notably improve sleep outcomes compared to no treatment. After adjusting for the CAT score, the use of ICS does not seem to impact sleep outcomes. CASIS100: COPD and Asthma Sleep Impact Scale, linearly transformed to a 0–100 scale, CAT: COPD assessment test, COPD: chronic obstructive pulmonary disease, ICS: inhaled corticosteroids, N: number of subjects, NT: no treatment, SD: standard deviation, T-ICS: COPD treatment scheme containing ICS, T-other: COPD treatment scheme not containing ICS.

The results from the CASIS questionnaire on the self-perceived quality of sleep of COPD patients correlated with (i) the severity of the disease, (ii) the classification of patients according to the ABCD system, and in particular to CAT assessment, (iii) the age and time-since-diagnosis of patients, and (iv) whether subjects were not receiving any COPD treatment or whether their treatment included inhaled corticosteroids or not. A multiple regression was run to predict sleep quality (as assessed by CASIS score) from CAT score (excluding its sleeplessness component) and the %predicted FEV1 post-bronchodilation. The multiple regression model predicted sleep quality, F_2,3451_ = 1397.5, p < 0.001, adj.R^2^ = 0.45. Both independent variables added statistically significantly to the prediction (p ≤ 0.001, Fig. 2). The predictive power of the multiple regression model increases if we use the total CAT score, without excluding its sleeplessness component (Supplementary Table 1). In particular, the higher the level of disease severity, the poorer the quality of sleep [F_3,3446_ = 34, p < 0.001], especially when moving from mild to moderate (as indicated by an increase in the overall CASIS score by 4.12 on average, p < 0.001), and from moderate to severe stages (as indicated by an increase in the overall CASIS score by 4.18 on average, p < 0.001). Moreover, patients belonging to either groups A and C (i.e., suffering from relatively less COPD symptoms in their daily routine with CAT scores < 10) experienced much better quality of sleep [F_3,3415_ = 330.16, p < 0.001], compared to those belonging to either groups B or D (i.e., suffering from relatively more COPD symptoms in their daily routine with CAT scores ≥ 10) (as indicated by a decrease in the overall CASIS score by 18.31 on average, p < 0.001). Both age and time-since-diagnosis had a positive correlation with the poor quality of night sleep (ρ = 0.122 and ρ = 0.104, p < 0.001) (Supplementary Fig. 4). Finally, ANOVA has identified an interaction between treatment strategy and quality of sleep [F_2,3451_ = 21.65, p < 0.001]; subjects without any treatment experienced poorer quality of sleep compared to treated subjects (as indicated by an increase in the overall CASIS score by 6.29 on average, p < 0.05) (Supplementary Fig. 3). Finally, following adjustments for symptom severity (i.e., CAT score), we demonstrate that, presence or absence of inhaled corticosteroids from the COPD treatment strategy does not affect sleep quality (Fig. 3, Supplementary Table 2).

## Discussion

The current study aimed at assessing the quality of sleep in COPD patients in the context of routine medical practice (office-based and hospital-based pulmonologists) in Greece, using the CASIS questionnaire and its correlation with various sociodemographic and clinical features. At least half of the study participants reported no or very infrequent problems in their night sleep. We have shown a mild association of sleep outcomes with age and disease duration, a clear association between self-perceived deterioration of sleep quality and COPD spirometric severity, as well as a strong association between poor quality of sleep and higher CAT scores. Furthermore, subjects without any treatment seem to experience poorer quality of sleep compared to treated subjects.

To our knowledge, this is the first study examining sleep quality in such a large sample of Greek COPD patients using this specific questionnaire. Since its development (in 2009), the use of CASIS questionnaire in studies is limited, so further evidence is needed to confirm its relevance in assessing sleep quality in COPD patients and its importance in guiding the clinical management of the disease. In Greece, a study by Ierodiakonou et al., using the UNLOCK dataset^18^, analysed the association of subjective sleep quality with disease status in COPD in primary care and found worse health status and COPD severity were associated with poor sleep quality in COPD patients^19^. A Greek version of CASIS questionnaire was used in the study translated via the back translation methodology and then the performance, cultural relevance and comprehensibility of the questionnaire was tested in a small group of patients^18^. This version of the CASIS questionnaire has been used by our study as well.

First of all, it should be noted that the features of our population sample show similarities to other large epidemiological studies conducted in Greece and abroad; there was a male predominance, the mean age of subjects lay between 65–70 years, most subjects were suffering from a spirometrically moderate to severe disease and mainly belonged to Groups B-D of the ABCD classification system^20–24^. Moreover, most patients were diagnosed with COPD for over 12 months prior study enrollment, LAMA/LABA combination was the most frequently prescribed regimen, a very high percentage of patients (exceeding 40%) were receiving more than one drug to control COPD symptoms, and finally, despite treatment, more than 40% of them reported exacerbations during the previous year.

In general, quality of sleep as assessed in SLEPICO study was better than that reported in other smaller clinical studies^19,25^, and the great majority of patients reported that COPD treatment had significantly improved their quality of sleep. The median value of the total CASIS score in our population of COPD patients is very close to the corresponding value of a similar COPD patient group, reported by Pokrzywinski et al.^15^, when they first introduced this measure of sleep quality. There are no studies on the general population, having used CASIS to report on quality of sleep. Therefore, attempts to compare the sleep quality issues on our study population with the age-matched general population would be risky. In our patient sample, sleep quality drops with age, a feature which we are unable to estimate to what extent it related to COPD. The prevalence of poor sleep quality is high among adults anyway (about a third), especially women. There is a direct relationship between age and deterioration in the quality of sleep of the general population, nevertheless this relationship appears to be more consistent in women^26,27^. More studies are required not only to establish a clearer relationship between ageing and the quality of sleep, but also to explore the psychometric properties of CASIS in the general population to be able to infer to what extent a sleep quality decline relates to COPD and not the ageing process per se.

Ierodiakonou et al.^19^ assessed sleep quality and associations of sleep impairment with health status, exacerbations, hospitalizations, ABCD classification, inhaler adherence, frailty, and sense of coherence, adjusting for age, gender, smoking status, and comorbidities. The study concluded that poor sleep quality is associated with COPD spirometric severity and worse health status. Moreover, Azkona et al.^28^ reported from their own observational, multicenter study, with similar aims as the SLEPICO, that patients with higher CAT score, degree of dyspnea, number of exacerbations, and lower FEV1% were more likely to have a high score on the CASIS scale. In accordance with these reports, our study confirms the association between self-perceived deterioration of sleep quality and COPD severity, as well as the strong association between poor quality of sleep and worse COPD health status as evaluated by a higher CAT score. Additionally, our study indicates that the age of patients as well as disease duration mildly affects sleep quality. On the contrary, no clear relationship appears to exist between quality of sleep and number of exacerbations; this inference is though indirect, deriving from the small differences on the quality of sleep between patient groups A and C, and between patient groups B and D in the ABCD classification system.

Another important finding of our work relates to the use of inhaled corticosteroids in the chronic management of COPD patients. As it is known, inhaled corticosteroids might affect the mode of the hypothalamic–pituitary–adrenal axis function^29^, especially when used in high doses, and this in turn, could change important components of sleep physiology; it has long been shown, for instance, that sleep duration, architecture and cortisol/corticotrophin secretion are interrelated^30^, cortisol increases accompany prolonged waking periods^31^ and changes in the underlying ultradian cortisol rhythm associate with changes in the self-perceived quality of sleep^32^. Our study results show that sleep outcomes do not vary among COPD patients under treatment, independent on whether this involves inhaled corticosteroids or not.

Limitations of the study include its observational nature, which limits its ability to draw any firm inferences on factors associated with changes in the quality of sleep, and the fact that the findings related to sleep improvements after starting the inhaled COPD treatment (which have been extracted by retrospective self-reports of the study participants) relay on individuals’ autobiographical memory, which is prone to random error but potentially also systematic bias. Another limitation was that adherence to COPD treatment was not recorded.

## Conclusions

Poor quality of sleep may further worsen the already diminished quality of life in COPD patients. Data from SLEPICO study reported that still many patients experience often or very often sleep problems. A regular check by healthcare providers of regarding quality of sleep or sleep disorders, followed by appropriate management, may contribute at improving the quality of life of COPD patients.

Our study provides a valuable record of the self-perceived quality of sleep of COPD patients in Greece, and its association with demographic and COPD-related, clinically relevant features of these patients. The study denotes that (i) increased age, (ii) prolonged disease duration, and especially (iii) CAT score ≥ 10, and (iv) severe COPD stage might act as important indicators for deterioration in the quality of sleep, with potential important limitations in daily life activities of COPD patients, thus urging potentially for further pharmacological interventions or modifications.

### Ethics approval and consent to participate

All methods were carried out in accordance with Good Clinical Practice, local laws, EU-Directive 2001/20, and the International Conference on Harmonization and the World Medical Association Declaration of Helsinki guidelines. Moreover, the experimental protocol was approved by the Institutional Review Board/ Ethics Committees of the participating Hospitals. Finally, an informed consent was obtained from all study participants.

## Supplementary Information

Supplementary Information.

## Data Availability

All data of the study have been presented in this manuscript and the corresponding supplementary materials.
