# Author Correction: Self-perceived quality of sleep among COPD patients in Greece: the SLEPICO study

**DOI:** 10.1038/s41598-022-06149-z

**Published:** 2022-02-01

**Authors:** Nikolaos Koulouris, Katerina Dimakou, Konstantinos Gourgoulianis, Nikolaos Tzanakis, Aggeliki Rapti, Mina Gaga, Niki Georgatou, Paschalis Steiropoulos, Christos Karachristos, Athena Gogali, Konstantinos Kalafatakis, Konstantinos Kostikas

**Affiliations:** 1 First Department of Pulmonary Medicine and Intensive Care Unit, National and Kapodistrian University of Athens, Medical School, 115 27 Athens, Greece; 2 5Th Respiratory Medicine Department, General Hospital for Chest Diseases of Athens “SOTIRIA”, Athens, Greece; 3 Department of Respiratory Medicine, Faculty of Medicine, University of Thessaly, BIOPOLIS, 41500 Larissa, Greece; 4 Department of Respiratory Medicine, University General Hospital of Heraklion, Medical School, University of Crete, 71003 Heraklion, Greece; 5 2Nd Respiratory Medicine Department, General Hospital for Chest Diseases of Athens “SOTIRIA”, Athens, Greece; 6 7Th Respiratory Medicine Department, Athens “Sotiria” Chest Diseases Hospital, Athens, Greece; 7 Iatriko Athinon, Areos 41, Paleo Faliro, Greece; 8 Department of Respiratory Medicine, Medical School, University General Hospital of Alexandroupolis, Democritus University of Thrace, Alexandroupolis, Greece; 9 Department of Pulmonary Medicine, General Hospital of Thessaloniki “Georgios Papanikolaou”, G. Papanikolaou Ave, 57010 Exohi, Greece; 10 Department of Informatics and Telecommunications, School of Informatics and Telecommunications, University of Ioannina, Arta, Greece; 11 Respiratory Medicine Department, University Hospital of Ioannina, Ioannina, Greece

Correction to: *Scientific Reports* https://doi.org/10.1038/s41598-021-04610-z, published online 11 January 2022

The original version of this Article contained an error in Funding information.

“The RELICO study has been sponsored by Menarini Hellas S.A.”

now reads:

“The SLEPICO study has been sponsored by Menarini Hellas S.A.”

The original Article has been corrected.

