# Supplementary material for: Self-perceived quality of sleep among COPD patients in Greece: the SLEPICO study
Source: Sci Rep. 2022 Jan 11;12:540. doi: 10.1038/s41598-021-04610-z (PMC8752730; doi:10.1038/s41598-021-04610-z)
Supplement: Supplementary file 1 — Supplementary Information. [file 41598_2021_4610_MOESM1_ESM.pdf]

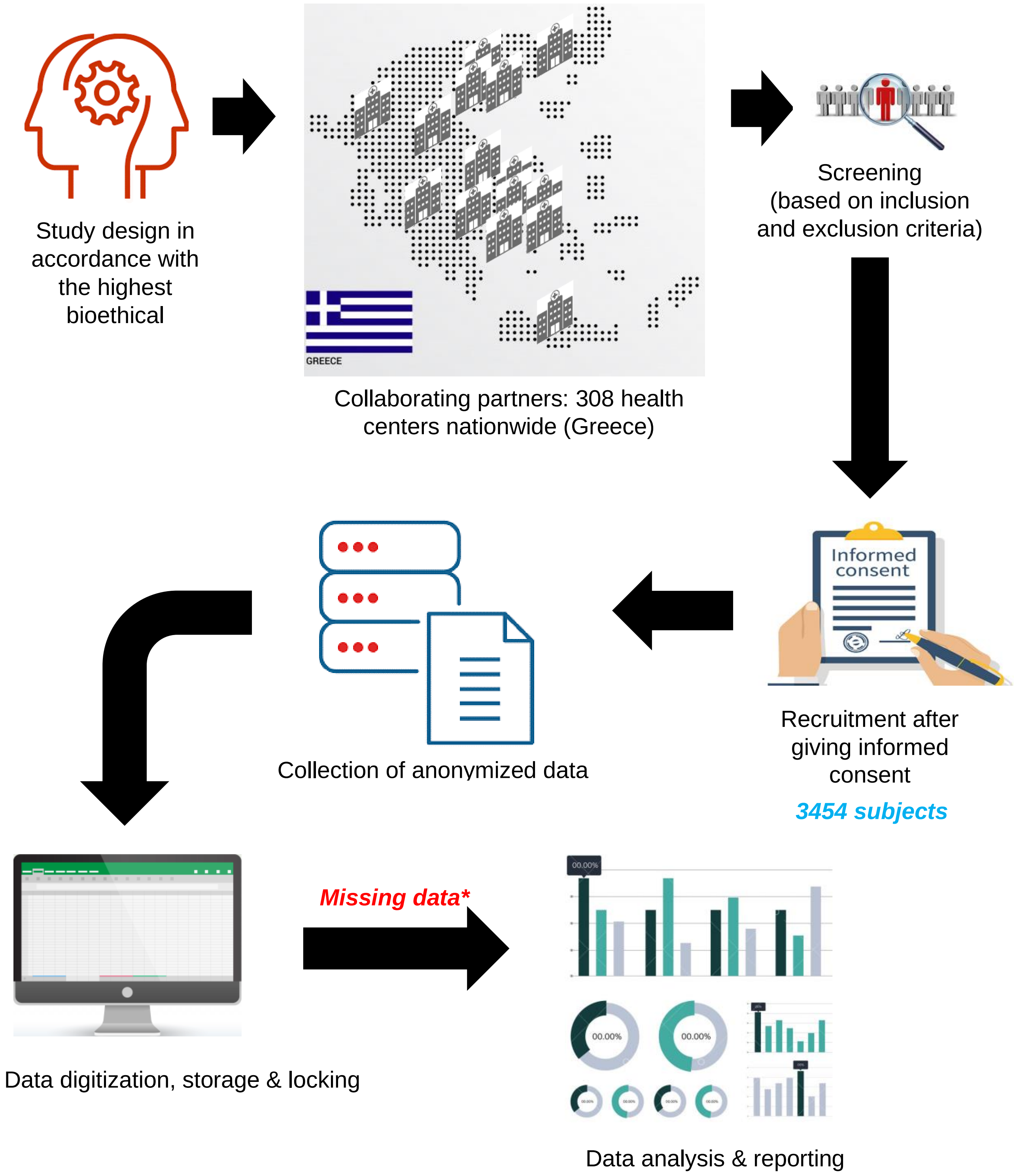

Supplementary Figure 1

Pipeline of the different stages of this cross-sectional observational study. The finalized database contains data from 3454 subjects. Ninety-seven different parameters (variables) were collected per subject. Various isolated missing values have been retrospectively identified in some of the subjects (*). These relate to either the subjects' or the medical doctors' negligence to complete all domains of the corresponding case report form, which occurred in a random manner, i.e., cannot introduce a systematic bias into the study.

Months since initial diagnosis (N=2127)

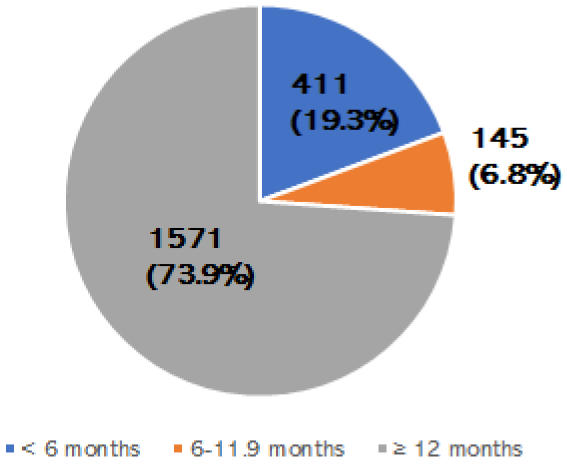

COPD family history (N=3454)

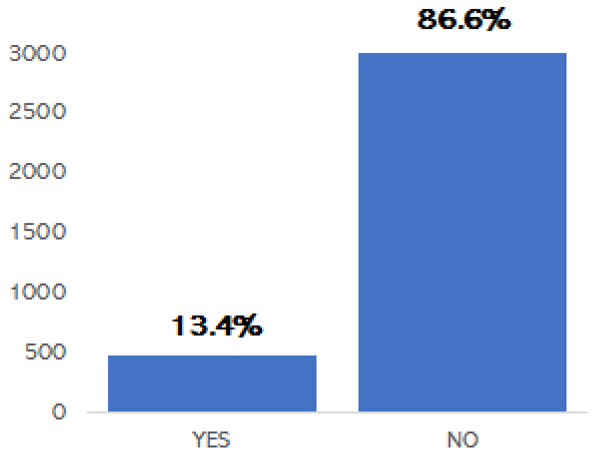

ABCD group (N=3452)

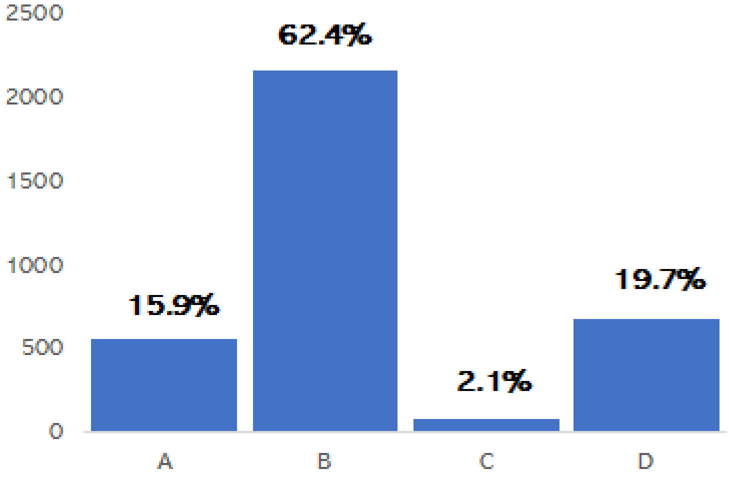

COPD management strategies*

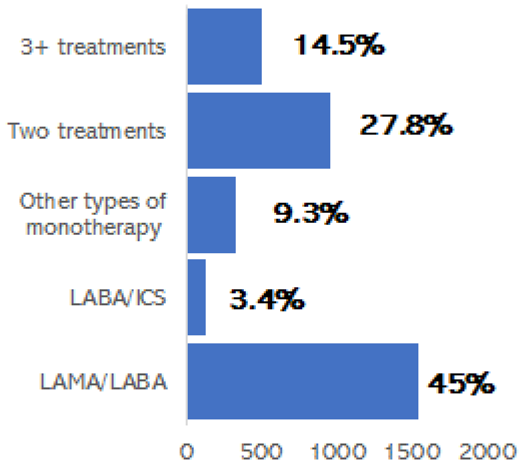

Pharmacological categories
% prescription in study sample

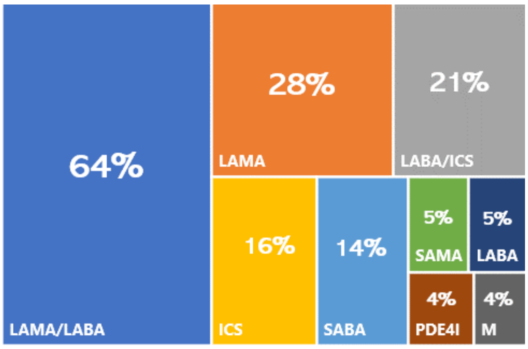

Comorbidities (N=3454)	
**Cardiovascular disease** *(CHF, IHD, arrhythmias, PVD, hypertension, stroke)*	1258 (36.4%)
**Metabolic disease** *(DM, metabolic syndrome)*	359 (10.4%)
**Psychiatric disease** *(Anxiety, depression)*	173 (5%)
**Gastrointestinal disease** *(Gastroesophageal reflux)*	122 (3.5%)
**Myoskeletal disease** *(Osteoporosis)*	78 (2.3%)
**Other** *(Including other pulmonary diseases, thyroid disease, BPH, dyslipidemia)*	358 (10.4%)

Supplementary Figure 2

COPD-related features of the study sample (summary statistics).

Group A: Low risk, low symptom burden. Patients have both a low exacerbation rate (0-1 exacerbation and no hospital admissions) and low symptom burden (CAT score < 10). Group B: Low risk, higher symptom burden. Patients have a low exacerbation rate (0-1 exacerbation and no hospital admissions) but high symptom burden (CAT score ≥10). Group C: High risk, low symptom burden. Patients have a high exacerbation rate (≥2 exacerbations annually or any number leading to hospital admission) but low symptom burden (CAT score < 10). Group D: High risk, higher symptom burden. Patients with both high exacerbation rate (≥2 exacerbations annually or any number leading to hospital admission) as well as high symptom burden (CAT score ≥10).

* 33 patients received no treatment for COPD (0.009% of the study sample)

BPH: benign prostatic hyperplasia, CHF: congestive heart failure, COPD: chronic obstructive pulmonary disease, DM: diabetes mellitus, IHD: ischemic heart disease, PVD: peripheral vascular disease

ICS: inhaled corticosteroids, LABA: long-acting β adrenoreceptor agonists, LAMA: long-acting muscarinic receptor antagonists, M: methylxanthines, PDE4I: PDE4 inhibitors, SABA: short-acting β adrenoreceptor agonists, SAMA: short-acting muscarinic receptor antagonists

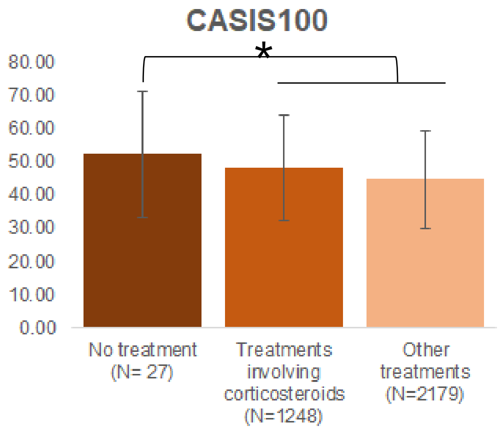

Sleep quality has been improved following initiation of the current inhalation COPD treatment (N=3025)	N	%
YES	2623	86.7
NO	402	13.3

Supplementary Figure 3

(Left panel) The overall CASIS score among COPD patients, categorized based on their type of treatment; either no treatment, or treatment strategy including inhaled corticosteroids, or treatment strategy not including inhaled corticosteroids. Notable differences have been specified between the no treatment group with the treatment groups.

(Right panel) Self-perceived, subjective notion of patients on whether sleep quality has been improved after initiating current inhalation COPD treatment (whatever this treatment might be).

CASIS100: COPD and Asthma Sleep Impact Scale, linearly transformed to a 0-100 scale, COPD: chronic obstructive pulmonary disease, N: number of subjects, SD: standard deviation

* $p < 0.05$

CASIS100	*B*	95% CI for *B*		SE *B*	β	R^2	ΔR^2
		LL	UL				
Model						0.50	0.50*
Constant	18.675*	16.894	20.456	0.908			
CAT	1.543*	1.490	1.596	0.027	0.720*		
$_{\%pred}FEV1_{postBD}$	0.041*	0.020	0.063	0.011	0.048*		

Supplementary Table 1

Multiple regression results for CASIS100, using as independent variables the %predicted FEV1 spirometric data post-bronchodilation (%predFEV1postBD) and the total CAT score, without excluding its sleeplessness component. The multiple regression model statistically significantly predicted sleep quality, $F_{2,3451} = 1718.9$, $p<0.001$. Both independent variables added statistically significantly to the prediction.

B: unstandardized regression coefficient, CASIS100: COPD and Asthma Sleep Impact Scale, linearly transformed to a 0-100 scale, CAT: COPD assessment test, CI: confidence intervals, COPD: chronic obstructive pulmonary disease, LL: lower limit, UL: upper limit, SE B: standard error of the coefficient, β: standardized coefficient, R^2: coefficient of determination, ΔR^2: adjusted R^2

* p ≤ 0.001

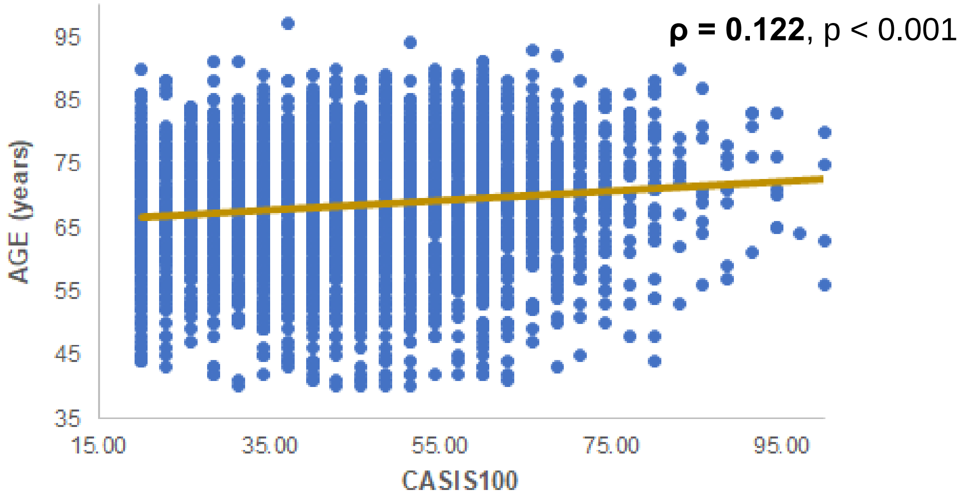

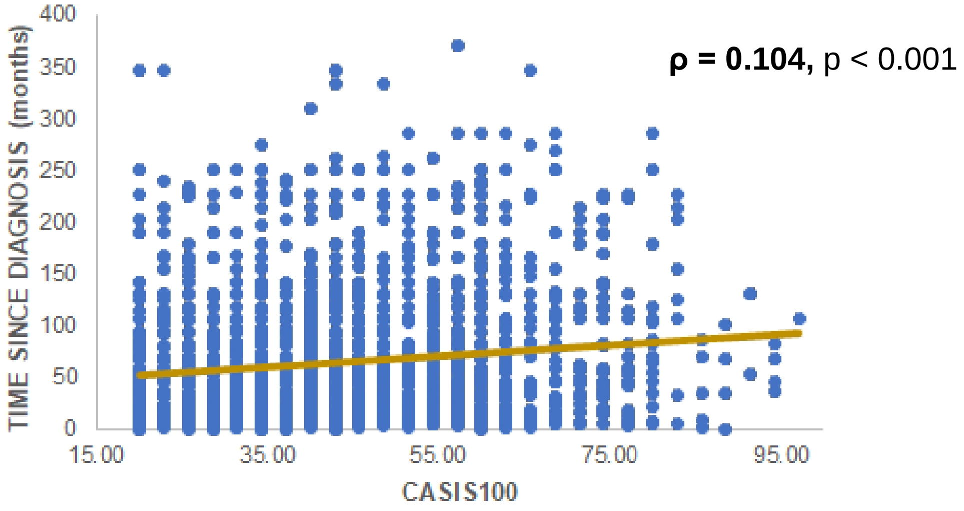

Supplementary Figure 4

Positive correlations between age and poorness in night sleep, as well as between time-since-diagnosis and poorness in night sleep. Spearman rank correlation coefficient ρ has been used. CASIS100: COPD and Asthma Sleep Impact Scale, linearly transformed to a 0-100 scale

	MEAN (± SD)			
	**CAT score**	**Age (years)**	**TsD (months)**	**Severity stage**
NT	14.85 (7.73)	66.48 (8.69)	123 (89.29)	Mild to Moderate
T-ICS	17.87 (7.28)	70.10 (9.22)	62.11 (63.55)	Moderate to Severe
T-other	15.16 (6.75)	67.61 (9.90)	66.42 (62.03)	Moderate to Severe

Supplementary Table 2

Summary statistics of different variables of the COPD patients included in this study (CAT score, age, time-since-diagnosis, severity stage), depending on which treatment category they belong to (no treatment, treatment including inhaled corticosteroids and treatment without corticosteroids). The patients in each therapeutic category vary on average in these parameters, especially in CAT scores. CAT: COPD assessment test, COPD: chronic obstructive pulmonary disease, ICS: inhaled corticosteroids, NT: no treatment, SD: standard deviation, T-ICS: treatment scheme containing ICS, T-other: treatment scheme not containing ICS, TsD: time since diagnosis
